# Berberine hydrochloride improves cognitive deficiency through hippocampal up-regulation of neurotrophins following inhalant self-administration of methamphetamine

**DOI:** 10.22038/IJBMS.2022.65053.14326

**Published:** 2023-01

**Authors:** Fahimeh Mohseni, Raheleh Rafaiee, Leila Rezaeian, Mohammad Niroumand Sarvandani, Hamid Kalalian Moghaddam

**Affiliations:** 1 Center for Health Related Social and Behavioral Sciences Research, Shahroud University of Medical Sciences, Shahroud, Iran; 2 Department of Neuroscience, School of Advanced Technologies in Medicine, Mazandaran University of Medical Sciences, Sari, Iran; 3 Student Research Committee, School of Medicine, Shahroud University of Medical Sciences, Shahroud, Iran; 4 Addiction Research Center, Shahroud University of Medical Sciences, Shahroud, Iran; 5 Department of Addiction Studies, School of Medicine, Shahroud University of Medical Sciences, Shahroud, Iran

**Keywords:** Addiction, Cognition, Substance abuse, Neurotrophic factors, Neuroprotective

## Abstract

**Objective(s):**

Chronic methamphetamine (METH) abuse is recognized as an important risk factor for cognitive impairment. A plant-based isoquinoline alkaloid, Berberine hydrochloride (BER), shows memory and cognition enhancement properties. Due to the aim of the present study which is to investigate the influence of BER administration on METH-induced cognitive deficits, we investigated neurotrophin signaling including brain-derived neurotrophic factor (BDNF) and glial cell line-derived neurotrophic factor (GDNF) as a possible mechanism by which BER exerts its cognitive improvement influences.

**Materials and Methods:**

In this experimental study, thirty-two male Wistar rats were randomly classified into four groups, including non-treated control, intubated control, METH-inhaled, and METH-inhaled + BER-intubated. Rats in the METH-inhaled group underwent METH inhalation for 14 days, and the BER-inhaled and BER-intubated rats were intubated (100mg/kg) for the following three weeks. A novel object recognition task (NORt) was carried out on days 36 and 37. Rats were sacrificed for histological preparations after the behavioral tests. Neurotrophic factors, including GDNF and BDNF, were evaluated by immunofluorescence staining in the hippocampus.

**Results:**

This experiment indicated a dramatic improvement in cognitive deficits associated with chronic METH abuse (*P*<0.001). Furthermore, a significant decrease in the expression of both neurotrophins, GDNF (*P*<0.001) and BDNF (*P*<0.001), was observed in the METH-inhaled group compared with the METH-inhaled group treated with BER and non-treated control group.

**Conclusion:**

Activation of neurotrophic factors after BER administration resulted in improvement of METH-induced cognitive deficits. Therefore, BER may be considered a promising treatment for METH users who experience cognition deficits.

## Introduction

Methamphetamine (METH), which is increasingly used worldwide, is the second most abused drug after cannabinoids (1). METH use is a serious risk factor for the development of cognitive problems. Severe cognitive deficits occur in at least 27% of users of ecstasy, an amphetamine derivative (2). Previous studies indicate that regular amphetamine use leads to learning and memory deficits and impairs decision-making (3). In addition, patients with chronic METH use perform poorly on tests of cognitive flexibility (4, 5). Moreover, METH use is related to dysfunction in attentional set-shifting (6), executive function (7), verbal recall, and recognition domains (8). In a recent study to screen for cognitive impairment in METH users, all cognitive sub-scores, including memory, attention, visuospatial functions, and verbal fluency were significantly impaired in the sample (9). The results of this study confirm the adverse effects of METH use on all domains of cognitive performance. Similar results have also been obtained in experimental studies. For instance, Bisagno *et al*. (2002) (10) and Schröder *et al*. (2003) (11) show that repeated administration of METH within a single day leads to profound deficits in a nonspatial task of recognition memory. Moreover, repeated moderate doses of METH damage monoaminergic terminals in the forebrain and non monoaminergic cells in the somatosensory cortex and impair performance in a novelty preference object recognition task (12). The essential forebrain structure for cognitive function is the hippocampus, which is highly sensitive to amphetamine derivatives (13). It is well known that dysfunction or damage to the hippocampal formation is closely associated with deficits in recognition memory (14). For example, amphetamine-induced cognitive impairment is accompanied by neurotrophic deficiency in the hippocampus (15). In addition, reports suggest that cognitive deficit related to amphetamine neurotoxicity may be associated with changes in brain-derived neurotrophic factor (BDNF) in the hippocampus in the METH-induced model of mania in rats (16). 

In other experimental studies, brain-derived neurotrophic factor (BDNF) was shown to decrease after repeated METH administration and to increase 30 and 90 days after psychostimulant withdrawal (17). In addition, another report shows that chronic amphetamine can decrease the concentration of glial cell line-derived neurotrophic factor (GDNF) and nerve growth factor (NGF) in the hippocampus (18, 19). 

It is also worth noting that a study found that serum BDNF levels were significantly and consistently lower in METH users during early withdrawal than in healthy controls (20). Overall, the results of the above studies indicate that METH abusers may exhibit severe neurotrophic dysfunction and impaired neuroprotective function after repeated use of METH.

Hospitalization, behavioral and cognitive techniques, and psychosocial methods are the only inadequate treatment alternatives for METH abuse (21). Although several herbal and nonherbal agents have been proposed and evaluated in animal and human models as therapeutics for METH abuse. However, to date, there is no Food and Drug Administration (FDA)-approved product (22). 

Berberine hydrochloride (BER) is an organic compound isolated from various medicinal herbs such as Berberis vulgaris that has multi-faceted defensive properties (23). One of the interesting protective properties of BER is the up-regulation of neurotrophic agents and their receptors by a yet unknown mechanism in the central nervous system (24-26). Considering the reports on the neuroprotective role of BER and the known negative effects of METH on cognition, this study aims to test the effect of administration of BER on the maladaptive function of neurotrophic factors after METH-induced neurotoxicity.

## Materials and Methods

***Animals***

This study was performed on male Wistar rats (200–250 g) provided by the Animal Institute (Shahroud, Iran). Before the experiments, the animals were given one week to acclimate to the new laboratory conditions. Five rats were housed in a cage and maintained on a 12-hr light-dark cycle (lights on between 7 am and 7 pm), with water and food available *ad libitum*. Housing was at controlled temperature (23 °C ± 2 °C) and humidity (60 ± 5%). The local ethics committee of Shahroud Medical University approved all procedures performed and complied with the recommendations of the NIH Guide for the Care and Use of Laboratory Animals.

***Experimental design***

The animals were divided into four groups, including: 

i. Non-treated control (n=8): No treatment (no BER or METH) 

ii. Intubated control (n=12): Gavage only, but no BER 

iii. METH-inhaled (n=7): 21 days of drug abstinence followed by 14 days of METH inhalation 

iv. METH-inhaled+ BER -intubated (n=12): METH Inhalation for 14 days plus BER (100 mg/kg) intubation during the 21-day withdrawal period.

**Figure 1:**
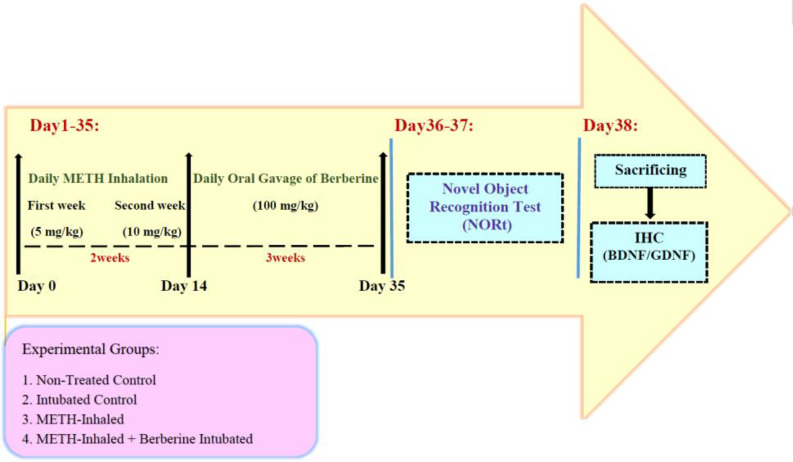
Schematic illustration of the experimental timeline

METH: Methamphetamine; IHC: Immunohistochemistry, BDNF: Brain-derived neurotrophic factor; GDNF: Glial cell line-derived neurotrophic factor

Two groups of rats (METH inhalation group, METH inhalation + BER intubation group) self-administered METH for 14 days in a preferred device used for such addiction animal models. Two concentrations were considered for the 1st and 2nd weeks of the addiction phase (1 mg/ml (5 mg/kg) and 2 mg/ml (10 mg/kg), respectively). A similar procedure was demonstrated in our recent work (27). During the 21-day withdrawal phase of METH, the administration of BER was accomplished through intragastric intubation (100 mg/kg) using a PE (polyethylene) 10 tube dipped in corn oil (28). The intubated control animals were intubated for 21 days, as was the BER-treated group, but only for 5 to 10 sec without fluid infusion. The non-treated group, which received no treatment or intubation, was weighed daily for two weeks. Then, on days 36 and 37, the novel object recognition task was performed. After the behavioral test, the rats were sacrificed for the histological studies. Neurotrophic factors, including BDNF and GDNF, were examined in the hippocampus by immunofluorescence. A schematic representation of the experimental procedure is shown in Figure 1.

***Drugs***

*METH-inhaled self-administration*

All rats in both METH-inhalation and METH-inhalation + BER -intubation groups were trained daily for 15 min with a self-administration device. Distilled water (DW) was used to dissolve METH (Sigma-Aldrich; Merck Millipore, M8750, USA). There were two levers in the device, one active and one inactive. When the inactive lever is pressed, nothing happens, but when the active lever is pressed, METH is received and a red diode lights up. In the self-administration part of the device, a syringe is installed that delivers 3 ml of the METH solution into a plastic microinjection tube, based on the amount specified by the researcher. Next to this part is a closed chamber into which a fan blows the vaporized METH solution, which the animal then inhales (27).

*BER intubation*

BER hydrochloride (Sigma-Aldrich; Merck Millipore, Germany) was dissolved in corn oil and administered through gavage (28).

***Behavioral test***

*Novel object recognition task (NORt)*

On day 36 of the experiment, NORt, a confirmed test of memory recognition in rodents, was performed; since rats prefer to explore new objects rather than familiar objects. Moreover, there is no positive or negative reinforcement in this test (29). NORt consists of three separate phases: habituation, familiarization, and testing phases. In the 1st phase, animals are placed in the center of the (60 × 60 × 60 cm) empty black box to explore it for five min. The habituation phase, in which two uniform objects were presented to each rat for 5 min, was performed the next day. The rat was then placed in its cage. The test sessions were conducted after a 60-min break between trials. In the test trials, a new object and a familiar object are presented for five min (30). Exploratory sniffing and touching behavior is observed during the first few seconds of the presentation of the new object. However, chewing and climbing on the object is not considered exploration. All objects were plastic products attached to the bottom of the cage to prevent dislocation. Before each test, the objects and the test apparatus were cleaned with ethanol (75% v/v) to prevent the formation of odorants (30).

The total exploration time of both objects in both habituation and test trials was calculated along with the discrimination index (DI).

DI = [time for exploration of the new object T (EN) ˗ time for exploration of the known object T (EF)]/(TEN + TEF), the discrimination index shows the difference in exploration time determined as part of the total time spent exploring the two objects during the test trial.

***Histological assessment***

*Tissue preparation and immunofluorescence staining*

To remove the brains, the animals were anesthetized on day 38 with a mixture of ketamine 100 mg/kg and xylazine 10 mg/kg by intraperitoneal injection to perform histological studies. Immediately, transcardial perfusion was performed in the anesthetized animals (a mixture of saline (0.9%) and paraformaldehyde (0.4%) in phosphate buffer (0.1 M) was used for perfusion). Then, the removed brains were paraffin-embedded to be fixed. 

The next step was coronal sectioning (7 μm) of the paraffin-embedded tissue based on the Paxinos atlas using a microtome (30). To determine neurotrophin activation, immunofluorescence staining after definition of BDNF and GDNF in 7-μm tissue sections was carried out. 

To hinder endogenous peroxidase activation, a solution of methanol and H_2_O_2_ (10%) was used for 10 min. Also, the sections were washed with Tris buffer solution. To retrieve antigen, for 11 min, brain sections were put in citrate buffer. The sections were fixed by adding 1% FBS in 0.3% Triton X-100, after the final wash with PBS. Samples were treated with polyclonal anti-BDNF antibody (PA1-18371, Thermo Fisher Scientific, USA) and a polyclonal anti-GDNF (PA1-1837159, 1:200, Thermo Fisher Scientific, USA). These primary antibodies were incubated overnight at 4 °C (dilution ratio, 1:100). For antigen detection, BDNF secondary antibody (FITC anti-rabbit IgG (ab6717, 1:200, Abcam, USA) and GDNF secondary antibody FITC-anti-rabbit IgG (ab97022, 1:200, Abcam, UK) were left at room temperature (for 0.5 hr). The visual determination of the number of BDNF-and GDNF-positive cells was performed by 400× magnification on each slide.

***Data analysis***

All data analysis was performed using the GraphPad Prism software (Prism for Windows, version 5.0, GraphPad Software Inc., San Diego, CA, USA). ANOVA was used to compare groups, and to assess the normal variable distribution Kolmogorov-Smirnov test was performed. Data are expressed as mean ± SEM. Dunnett’s T3 test (inhomogeneous variances) or Scheffe’s *post hoc* test (homogeneous variances) was used to assess differences. The significance level was considered at *P*≤0.05. 

## Results

***Berberine decreased METH-induced cognitive impairment***

**Figure 2:**
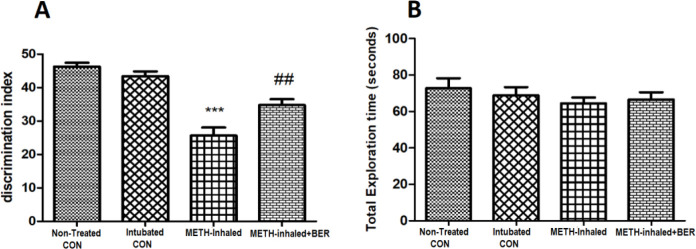
Discrimination index (A) and total exploration time (B). The discrimination index shows the difference in exploration time, measured as ( novel object - familiar object)/(exploration time familiar object + novel object). Total exploration time indicates the total exploration time for the two objects during the test sessions and the habituation phase, which is calculated together. Triple asterisks (***) are significantly different from the nontreated control group (*P*<0.001). Data are expressed as mean±SEM

Statistical analysis showed that DI (discrimination index) was lower in the METH-inhaled group of rats compared with the non-treated control group (*P*<0.001). DI was significantly decreased in the METH-inhaled group of rats compared with the non-treated control group (*P*<0.01, Figure 2A). In addition, Figure 2B showed no significant difference in total exploration time compared with the METH-inhaled group. This finding shows the cognitive impairment that occurred in METH-inhaled rats. In contrast, METH-inhaled rats treated with BER explored more novel objects and the DI increased, suggesting that BER has a protective effect on the cognitive deficits caused by METH.

***Berberine improved hippocampal BDNF and GDNF expression deficiency caused by self-administration of METH***

**Figure 3:**
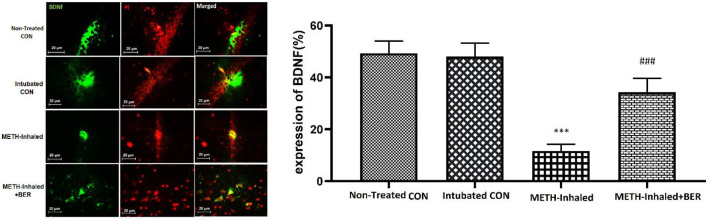
BDNF immunofluorescence staining in rat hippocampus. (A) Staining of nuclei with antibodies against BDNF and DAPI and their merge from every group (magnification, ×400). (B) Amount of positive reaction in each group. Scale Bars: 20 µm. The ratio of BDNF-positive neurons is shown as mean±SEM (n=4 per group); Triple asterisks (***) are significantly different compared with the non-treated control group (*P*<0.001). Triple hash (###) significantly different compared with METH-inhalation group (*P*<0.001)

BDNF: Brain-derived neurotrophic factor; METH: Methamphetamine; DAPI: 4′,6-diamidino-2-phenylindole

**Figure 4:**
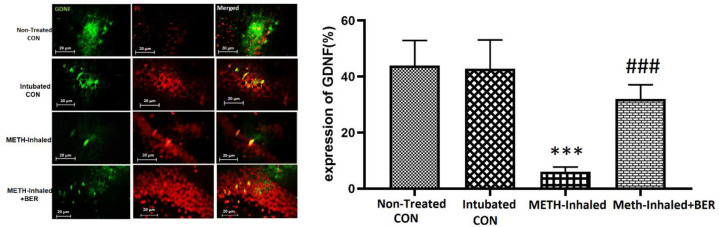
GDNF immunofluorescence staining in rat hippocampus. (A) Staining of nuclei with antibodies against GDNF and DAPI and their merge from every group (magnification, ×400). (B) the number of positive reactions in each group. Scale Bars: 20 µm. Ratio of GDNF-positive neurons is shown as mean±SEM (n=4 per group); triple asterisks (***) are significantly different from the non-treated control group (*P*<0.001). Triple hash marks (###) significantly different compared with METH -inhaled group (*P*<0.001)

GDNF: Glial cell line-derived neurotrophic factor; METH: Methamphetamine; DAPI: 4′,6-diamidino-2-phenylindole

Analysis of immunohistochemical data showed significantly lower expression of GDNF- and BDNF-positive cells in the METH-inhaled group compared with the non-treated group of the control group (*P*<0.01). Nevertheless, the percentage of BDNF-positive cells was significantly higher in the BER-treated METH-inhaled group (*P*<0.001) than in the METH-inhaled group (Figure 3). Moreover, the results showed that the METH-inhaled group expressed low levels of GDNF compared with the METH-inhaled group treated with BER (*P*<0.001, Figure 4). In conclusion, the results of this section show that BER increases the expression of GDNF and BDNF in the hippocampus after METH-induced neurotrophic deficiency.

## Discussion

This study demonstrated that METH-induced cognitive impairment was significantly improved by administration of BER, possibly by promoting the expression of neurotrophins in the hippocampus, including BDNF and GDNF. Of note, our findings are consistent with evidence showing that consumption of METH leads to cognitive impairment and tissue damage (31). Moreover, our results are consistent with recent studies in animal models indicating the beneficial neuroprotective effects of BER against various disorders of the central nervous system, including schizophrenia (32), Alzheimer’s disease (33, 34), depression (35, 36), anxiety (37), brain stroke (38), Parkinson’s disease (39), and addiction. Alavijeh *et al*. (2019) applied the two-bottle choice paradigm METH and treated it with 100 mg/kg/day BER (during withdrawal) via oral gavage for three weeks. BER reduced locomotor activity and anxiety-like behaviors (elevated plus maze test) and drug preferences in the two-bottle choice model (one week). In addition, BER increased the number of oxytocin receptors in the hippocampus and nucleus accumbens (40, 41). Our previous research results show that administration of BER to inhaled METH-addicted rats via modulation of neuroinflammation (NF-Κb, TLR4, Sirt1, and α-actin) improves anxiety-related behavior and decreases relapse (in the conditional place preference task) (40). The other previous study also concluded that BER improved cognitive dysfunction (spatial learning and memory in the Morris Water Maze and passive avoidance task in the Shuttle Box) caused by consumption of METH and brain inflammation through decreased activation of caspases-3, higher percentages of Ki67 expression, and increased GFAP expression (42). 

In humans, cognitive functions are often achieved through spoken or written language. Because this ability is not present in animals, different types of mazes and apparatuses have been developed depending on which aspect of cognition is desired (43). One common procedure called NORt is a hippocampus-dependent test that examines nonspatial recognition memory in murine (44). In NORt, rodents consider two identical objects and then, after a delay, discriminate between the familiar object and a novel object. NORt measures recognition memory, which is necessary for the development of memory capacity (45, 46). 

Experimental studies show that the time spent by the METH-treated group exploring the new object is dramatically less than the total time spent exploring both objects compared with the intact control group (47-49). Our results in the present study suggest that BER has a strong therapeutic effect on METH-induced cognitive impairment in NORt. The current results are consistent with those of Shi *et al*. who described an improvement in recognition memory in NORt after administration of BER in septic mice (50). In another study consistent with our experiments, it was found that 6-hydroxydopamine-induced impairment of memory in object recognition was reversed by oral administration of BER in rats with Parkinson’s disease (51). 

In addition, we found that BER-induced improvement in recognition memory after consumption of METH was related to the raised expression of BDNF and GDNF in the hippocampus. In general, neurotrophins are one of the known biomarkers with widespread cognition-enhancing properties (52, 53). Studies using genetic and pharmacological alterations of neurotrophin levels in the brain also suggest that performance in various cognitive domains can be enhanced by the increased availability of neurotrophins and impaired by their depletion (53). One of the first seminal studies on this topic, conducted in 1999, showed that aspects of cognition, including spatial learning and memory, improved in cognitively impaired aged rats after intracerebroventricular administration of GDNF, whereas no brain regional correlates were analyzed (54). Later studies have shown that GDNF can restore cognitive abilities after local administration in the hippocampus (55). In addition, previous studies on the role of BDNF in cognitive processes focused on tasks that depend on the hippocampus and showed that memory consolidation and acquisition are related to increases in BDNF mRNA expression and activation of the TrkB receptor in this area of the brain (56). Conversely, hippocampus-specific deletion of the BDNF gene or local injection of anti-BDNF antibodies results in specific blockade of hippocampus-dependent functions, leading to memory and cognitive deficits (56, 57). The present study confirms previous findings that the cognitive consequences of administration of METH are associated with neurotrophic deficiency in the hippocampus (57). Despite the cognitive enhancing effects of neurotrophic factors, the passage of high molecular weight proteins such as BDNF and GDNF through the blood-brain barrier (BBB) is negligible (58-60). Therefore, systematic administration of neurotrophins as a therapeutic tool is not effective in improving the cognitive effects of METH. On the other hand, direct administration to the brain requires surgical intervention (61), which makes it impossible to consider it a therapeutic agent for humans. The use of compounds that can stimulate the production of neurotrophic factors can potentially be used clinically if there are no side effects and no pharmacokinetic limitations. Recently, a 2021 study used a fluorescently labeled BER derivative to study subcellular processes by fluorescence imaging and found that BER can readily penetrate the BBB and exert its protective effects on neurons (62). One of the most important neuroprotective effects of BER in the brain is its ability to up-regulate neurotrophic substances and their receptors. For example, Yang and colleagues have shown that BER can exert neuroprotective effects in rats that have suffered cerebral ischemia by reducing the rate of apoptosis by the BDNF-TrkB- PI3K/Akt pathway (62). Furthermore, in a scopolamine-induced memory loss model, BER reduces the release of proinflammatory cytokines and increases the formation of neurotrophic factors, including GDNF, BDNF, and cAMP-response element-binding protein (CREB), a transcription factor coupled to BDNF activation (63). 

In this regard, some studies indicated that BER can activate the neurotrophic BDNF/TrkB signaling pathway in the hippocampal area of the brain (25, 64). Moreover, chronic treatment with BER attenuated depression in mice, which was mediated by up-regulation of BDNF expression in the hippocampus (24). Moreover, treatment with BER in combination with resistance training alleviated the neurological dysfunctions in the hippocampal area of the brain of diazinon-treated rats by up-regulating ERK and TrkB (24). In addition, BER has been shown to protect against cognitive impairment by stimulating PKC/ ERK /CREB, further increasing the expression of BDNF and inhibiting the downstream PKC effector GSK3β in quinolinic acid-injected mice (mouse model of Huntington’s disease)(65). Oliveira *et al*. (2019) also reported that oral administration of 50 and 100 mg/kg BER for 21 days was effective in preventing damage to recognition memory, as assessed by NORt, in an animal model of Alzheimer’s disease by streptozotocin induction in rats (66).

Double hash marks (##) were significantly different compared with METH-inhaled group (*P*<0.01)

METH: Methamphetamine; BER: Berberine; CON: Control

## Conclusion

In the current study, we found that activation of neurotrophic factors after administration of BER resulted in improvement of METH-induced cognitive deficits. Therefore, BER may be considered a promising treatment for METH-users who continue to experience memory and cognition deficits after quitting METH.

## Authors’ Contributions

HKM Conceived, planned, and supervised the experiments; FM and LR Performed material preparation, data collection, and analysis; RR Wrote the first draft of the manuscript, and all authors commented on previous versions of the manuscript. All authors read and approved the final manuscript.

## Funding

This work as a PhD dissertation (Project No. 9648) was supported by Shahroud University of Medical Sciences, Iran. The Local Ethics Committee affiliated with Shahroud University of Medical Sciences has approved this study (Registration code: IR.SHMU.REC.1396.30).

## Conflicts of Interest

The authors have no conﬂicts of interest to declare.
